# Miscellany

**Published:** 1902-06

**Authors:** 


### Miscellany.

To varnish the Inside of Cavities without touching the Margins with the Varnish.—Use an instrument having a swollen end, either bulbous or fan-shaped. The little tool used by jewellers for oiling watches answers the purpose. It works on the principle of attraction. The swollen end holds by attraction the drop of varnish, thus preventing it from running up the shank. To further aid in this, do not dip it deep into the varnish. When touched to the tooth, the superior attraction of the larger body causes the liquid to leave the instrument and spread over the walls of the cavity.—*Pacific Medical Journal.*

[The attraction in this case is nothing more than that of adhesion, and the size of the bodies has no practical influence.—Mc.]

---

Hydrogen Dioxide for Powder-Stains.—Recently Dr. J. N. Rhoads reported a case in which powder-stains had been removed by the use of hydrogen dioxide. In a recent issue of the *American Medicine* he published his experience of a single case. (A synopsis was noted in the *Monitor,* April 15.)

In an issue of the same journal, June 1, Dr. C. R. Clark, of Youngstown, Ohio, reported a similar case in which the face was black with stains and embedded particles of powder. Applications were made with lint saturated with glycerin, one part, and hydrogen dioxide, three parts.

At the end of two days all discoloration had disappeared.—*Medical and Surgical Monitor.*

---

Care of the Teeth.—The following are a few thoughts taken from an editorial in the May number of *Medicine* for 1901:

In no department of medical science has bacteriology made greater progress than in relation to the disorders of the teeth. Hygiene of the teeth, like that of the body in general, is comprehended in the word cleanliness. Additional importance is given to the matter by the great frequency of so-called pyorrhœa alveolaris, or what is better known as gingivitis.

The hygiene of the mouth has not been taught as earnestly nor as enthusiastically as it should be, notwithstanding the fact that the general health is largely dependent upon the digestion, and the latter upon the ability to masticate the food.

In addition, the suppurative condition which we have spoken of as gingivitis undoubtedly leads to direct infection of the alimentary tract. Numerous cases are on record in which grave digestive disturbances were at once relieved by the cure of the suppurative inflammation of the gums.

[There is no doubt that many disorders of a constitutional nature are produced by the absorption of the products of infection and suppurative inflammation from the oral cavity.

The medical profession concerns itself mainly with the cure rather than the prevention of disease, and this subject has not been given the consideration that its importance warrants. Much disease would be prevented by proper attention to oral hygiene. May not gout and rheumatism more often be caused by pyorrhœa alveolaris than be the cause of this pyorrhœa.—Mc.]

---

Use of Preservatives for Solutions of Suprarenal Extract.—When the extract of suprarenal gland was first introduced into medicine as a valuable constrictor of those blood-vessels with which it was brought into contact, much difficulty was found in preserving solutions of it from decomposition and change, and various substances were added to it to prevent alterations taking place in its character. Boric acid and similar preservatives were employed without much success; and recently in the *New York Medical Journal* of March 9, 1901, Oppenheimer records the results which he has obtained by adding resorcin in the strength of one per cent. to the solutions of suprarenal gland with the object of preserving them. He states that by this means he has been able to counteract all deteriorating influences. As a matter of fact, if suprarenal gland in its original form is employed, we believe that the best preservative to be added to it is chloretone, which is not only an anæsthetic but an antiseptic in its influence upon the mucous membranes, and which certainly seems to increase rather than to decrease the activity of the extract. With the advances which are being made, however, in the preparation of suprarenal gland for medicinal use, it would seem probable that even so useful a preservative as chloretone will eventually not be required, and there is now upon the market, under the name of adrenal*in chloride,* an active principle of suprarenal gland which in even so diluted a solution as 1 to 20,000 exerts a physiological effect, and which when dissolved in normal salt solution in the strength of 1 to 5000 is a most efficient local vasoconstrictor.—*Therapeutic Gazette.*

---

Gold Fillings in the Mosaic Period.—The *Detroit Medical Journal* culls from the *Red Cross Notes* the following:

The oldest medical writing which has been discovered is the Ebers Papyrus in the University Library at Leipsic containing over one hundred pages, in hieratic characters. On one of the leaves is evidence that this manuscript dates from at least 1550 B.C., or before the book of Exodus was written. Evidence is not wanting also to show that this is only a copy of a much earlier work. The entire compilation is claimed to be hermatic,—*i.e.,* inspired.

More than seven hundred materials from the mineral, vegetable, and animal kingdoms are mentioned as medicaments, and the mixtures prescribed are generally complex, ten or twelve ingredients entering thereinto. It is also worthy of note that cotton is mentioned as an application to wounds, and even in that early age teeth were filled with gold.

---

Fastening a Matrix with Oxyphosphate.—When necessary to use a matrix, I fasten it as follows: Take quick-setting oxyphosphate of zinc and place it around the soft copper strip which has been adapted to the cavity. Then in five minutes you can mallet against it from any direction without danger of loosening it.—J. N. Crouse, *Dental Review.*

[It was in the discussion of a paper entitled "Matrices for Approximal Gold Fillings" that brought out the above method of fastening a matrix. The unyielding nature of oxyphosphate cement around a matrix would prove undesirable and be sure to result in poor margins to the gold filling. If the soft copper matrix be fastened around the tooth with several turns of a ligature, it will give enough to allow the gold to come over the margins of the cavity to make a perfect filling.—McClain.]

---

Use of Cork between the Teeth while malleting.—If you would have your patient thank you, use a piece of cork, trimmed square, between the teeth when inserting a gold filling. The impact of the mallet blows is less severe on a tooth thus supported by a cushion of cork.—*Dental Hints.*

---

Collodion as a Model Varnish.—Dr. D. H. Payne, in the *Items of Interest,* recommends collodion as a better varnish than sandarach for varnishing plaster models previous to packing the rubber for vulcanizing. It does not adhere to the vulcanized plate and leaves a smooth and hard surface. To separate a model from a plaster impression varnish with thin collodion and dust the surface with talcum powder, brushing off all loose powder.—*Dental Register.*

---

A New Impression Material.—John Girdwood, in a paper read before the American Dental Society of Europe, describes the advantages of sealing-wax over plaster and modelling compound for taking partial impressions and impression and bite in crown- and bridge-work. When the bite is close, plaster frequently fractures so badly that it is next to impossible to piece it together. Impression compound, on the other hand, cannot be made sufficiently hard and unyielding as not to bend or drag during its removal. He claims that sealing-wax combines the properties of both in addition to having special advantages of its own.

He states that he has used sealing-wax as an impression material in crown- and bridge-work for several years. There are several varieties of sealing-wax. The highest grade is composed of pure lac and vermilion, and the lowest grade of rosin and cheap coloringmatter. It is the lac which makes it valuable to the dentist. He says, "A trial of the improved wax at once showed me I had obtained a suitable material for my purpose, but wishing to see whether it could be further perfected, I stated my requirements to the manufacturers, stipulating for a material which would soften readily, remain plastic until nearly cold, and retain that condition long enough to enable me to take any impression, or impression and bite combined. In addition to the lac and vermilion, some other agent was added to give additional softness. An alteration in the proportion of the ingredients enabled them to furnish me with a material which I found all that could be wished."—*Dental Review.*

---

TEST FOR ADULTERATED ZINC SALTS.—The zinc salts most likely to be unsatisfactory are the oxide and the chloride. The oxide should be an impalpable powder free from grit. The color is a secondary consideration, and may be disregarded. The bulk of the zinc oxide of commerce is made for paints, in which whiteness is the first consideration.

The chloride frequently contains oxychloride, which is insoluble in water. Dissolve a little of the sample and the presence of this impurity will be shown.—*Australian Journal of Pharmacy.*

